# Enhanced Vasoconstriction Mediated by α_1_-Adrenergic Mechanisms in Small Femoral Arteries in Newborn Llama and Sheep Gestated at Low and High Altitudes

**DOI:** 10.3389/fphys.2021.697211

**Published:** 2021-08-04

**Authors:** Fernando A. Moraga, Roberto V. Reyes, Germán Ebensperger, Vasthi López, Aníbal J. Llanos

**Affiliations:** ^1^Laboratorio de Fisiología, Hipoxia y Función Vascular, Departamento de Ciencias Biomédicas, Facultad de Medicina, Universidad Católica del Norte, Coquimbo, Chile; ^2^Laboratorio de Fisiología y Fisiopatología del Desarrollo, Programa de Fisiopatología, ICBM, Facultad de Medicina, Universidad de Chile, Santiago, Chile; ^3^Centro Internacional de Estudios Andinos (INCAS), Universidad de Chile, Santiago, Chile

**Keywords:** newborn llama, newborn sheep, sea level, chronic hypoxia, femoral vasoconstriction, noradrenaline, phenylephrine

## Abstract

The authors previously demonstrated that newborn llama (NBLL) express high levels of α_1_ adrenergic receptors, which provide a potent vasoconstriction response when compared with newborn sheep (NBSH) gestated at sea level. However, data regarding the impact of chronic gestational hypobaric hypoxia on α-adrenergic vasoconstriction in the neonatal life has not been studied. We evaluated if gestation under chronic hypobaric hypoxia modifies α_1_-adrenergic vasoconstrictor function in NBLL and NBSH. We compared the vasoconstrictor response induced by potassium and α-adrenergic stimuli in isolated small femoral arteries of NBLL and NBSH gestated at high altitude (HA; 3,600 m) or low altitude (LA; 580 m). The maximal contraction (*R*_MAX_) and potency (EC_50_) to potassium, noradrenaline (NA), and phenylephrine (PHE) were larger in HA-NBLL than LA-NBLL. *R*_MAX_ to potassium, NA, and PHE were lower in HA-NBSH when compared with LA-NBSH and potency results were similar. Competitive blockade with prazosin showed that RNLL LA/HA have a similar pA_2_. In contrast, NBSH had increased pA_2_ values in HA when compared with LA. Finally, small femoral arteries denudated or treated with LNAME in LA and HA lacked NO or endothelium participation in response to PHE stimulation. In contrast, NBSH displayed that denudation or blockade with LNAME support NO or endothelium participation in response to PHE activation. In conclusion, HA chronic hypoxia enhances α_1_ adrenergic receptor activity in small femoral arteries in NBLL to a higher degree than NBSH, implying a higher vasoconstriction function.

## Introduction

When humans and animals are exposed to high-altitude (HA) environments, the low barometric pressure decreases the amount of oxygen in the body (Conkin and Wessel, 2008). Chronic hypobaric hypoxia conditions likely exerted a selective pressure on those species with ancestry at HA, which allowed them to develop cellular and physiologic defense strategies to live under low atmospheric PO_2_ (Monge and León-Velarde, 1991; Llanos et al., 2007). Camelids are dwellers of the Andean plateau that have a wide distribution between 3,500 and 5,000 m above sea level, and evidence supports an ancestry of over 2 million years in this low PO_2_ environment (Stanley et al., 1994). Sheep (*Ovis aries*) and llama (*Lama glama*) have been compared to evaluate how species manage to dwell at HA; the former as a paradigm of acclimatization, with only 500 years in highlands and the latter of adaptation. Both species have been compared in studies of perinatal physiology and cardiovascular adaptations to chronic hypoxia (Llanos et al., 2003, 2007; Reyes et al., 2020).

The llama is a camelid species that inhabits the Alto Andino plateau. They are often studied for their notable physiological adaptations to hypoxia (Meschia et al., 1960; Banchero and Grover, 1972; Braunitzer, 1980; Llanos et al., 2007), that is the hemoglobin mutation, allowing a higher hemoglobin oxygen affinity (Braunitzer, 1980; Moraga et al., 1996). Additionally, studies performed during fetal life show strong vasoconstriction when the fetus is submitted to acute hypoxia. This response is induced by the release of catecholamines, neuropeptide Y (NPY), and other vasoconstrictors (Giussani et al., 1996, 1999; Reyes et al., 2020). α-Adrenergic vasoconstriction is higher in sea level fetal llama than fetal sheep. It is also critical for blood flow redistribution and survival during acute hypoxia since α-adrenergic blockade elicited death in fetal llama but not in fetal sheep (Giussani et al., 1999). However, in chronically hypoxic fetal sheep, α-adrenergic peripheral vasoconstriction is also enhanced and is critical for survival (Block et al., 1984; Giussani et al., 1996, 1999). We previously demonstrated that llamas living at sea level for several generations deliver newborns with a high femoral vasoconstrictor response to acute hypoxia compared with their sheep counterparts. Also, sea level newborn llamas (NBLL) have greater femoral sensitivity and maximal contraction to norepinephrine and phenylephrine (PHE) than newborn sheep (NBSH). This sensibility was corroborated by competitive inhibition experiments that showed higher prazosin affinity in NBLL than NBSH. This indicates the presence of one high-affinity adrenoceptor, which is consistent with the greater α_1B_-adrenoceptor transcript expression observed in the small femoral arteries of NBLL compared with NBSH (Moraga et al., 2011). In addition, α_1_-adrenergic receptor activation by PHE may be counterbalanced by simultaneous NO release from the endothelium in neonatal sheep but not in neonatal llama at lowland (Moraga et al., 2011). However, the effect of chronic hypoxia on the α-adrenergic vasoconstrictor tone has not been compared between species with or without HA ancestry (Longo and Pearce, 1998; Goyal et al., 2014). We hypothesize that HA chronic hypoxia enhanced femoral α-adrenergic vascular reactivity in NBLL but not in NBSH. For this, we used pharmacological approaches to test the role of adrenergic receptors in small femoral arteries by comparing the responses of llama (NBLL) and sheep neonates (NBSH) gestated at low altitude (LA) and HA.

## Materials and Methods

All the procedures of animal care, maintenance, and experimentation were performed in accordance with the UK Animals (Scientific Procedures) Act, 1986, and the American Physiological Society “Guiding Principles for Research Involving Animals and Human Beings” (American Physiological Society, 2002), and approved by Faculty of Medicine Ethics Committee of the University of Chile.

### Animals

We studied eight NBSH and seven NBLL s gestated, born, and raised at the University of Chile farm, Santiago, located at 580 m above sea level; and six NBSH and six NBLL, gestated, born, and raised at Putre Research Station, International Center for Andean Studies (INCAS), University of Chile, located at 3,600 m above sea level (see Supplementary Table). The ancestry of HA maternal sheep has been estimated at over 50 generations (Herrera et al., 2007) and around 2 million of years for maternal llamas (Stanley et al., 1994). The llama and sheep mothers and their newborns were maintained with access to food and water *ad libitum* in an open yard. The shed time of both llamas and sheep was programmed so that all deliveries occurred in the same spring–summer season. The installed experimental station is in Putre, which is at 3,500 m. The measured values indicate a low relative humidity close to 20%, with thermal oscillations of 5°C at night and 16°C during the day in the spring–summer season. All the animals used for the study of small arteries were healthy and showed the expected postnatal growth for the altitude where the experiment was performed.

### *Ex Vivo* Small Vessel Wire Myography

All the animals underwent euthanasia using sodium thiopentone (1 g I.V.). We dissected from deep femoral artery where small arteries of approximately 300–350 μm diameter and 1.8–2.0 mm length were cut and placed in ice-cold saline (see Supplementary Table). Arterial rings were mounted in a myograph to perform studies of isometric force (610M, Multimyograph, Danish Myotechnology, Aarhus, Denmark) by continuously recording tension using a data acquisition system connected to a computer (Powerlab/8SP; AD Instruments) (Moraga et al., 2011). The small femoral arteries were evaluated with (+E) or without (–E) endothelium. To eliminate the endothelium from the femoral arteries, a strand of human hair was gently rubbed backward and forward through the lumen of the vessel (Auer and Ward, 1998). To ensure the endothelium was denudated, we evaluated the response to the endothelium-dependent vasodilator ACh (10^−5^ M) in arteries, previously contracted with 10^−5^ M PHE. All endothelium-denudated rings lacked relaxation when exposed to the ACh bath, indicating that denudation was successful. Afterward, all small femoral arteries (+E or –E) were incubated in Krebs Ringer Bicarbonate (KRB) at 37°C and gassed (95% O_2_ and 5% CO_2_). After a period of incubation (1 h), the optimal diameter was determined for each artery by stretching the artery rings in a stepwise manner until a tension equivalent to the physiological transmural pressure was obtained (Mulvany and Halpern, 1997). In this condition, a dose of 62.5 mM of potassium chloride was added to promote a contraction dependent on the degree of stretch until it gave the maximal vascular response (Moraga et al., 2011; Castillo-Galán et al., 2016). Concentration-response curves (CRC) were performed for potassium chloride by adding 6.25–125 mM of KCl, α-adrenergic agonist NA, and α_1_-adrenergic agonist PHE, with concentrations ranging from 10^−10^ to 10^−3^ M as described previously (Moraga et al., 2011). The participation of endothelium in modulating the vasoconstriction mechanism was evaluated through concentration-response curves for PHE in intact (+E) and endothelium-free (–E) femoral arteries, either in the presence or the absence of the nitric oxide synthase (NOS) inhibitor L-NAME (10^−5^ M). The relative affinity of prazosin was determined by performing concentration response curves to NA in preincubated vessels for 30 min with prazosin (α_1_-adrenergic receptor blocker with doses of 10^−9^, 10^−8^ and 10^−7^ M). Each curve was evaluated in triplicate. We collected atleast 12–24 rings of small femoral arteries per animal to carry out the different assays. After finishing one curve, each artery was maintained at rest, with KRB, for at least 30 min, changing KRB solution every 10 min. Before initiating a new curve, contraction induced by 62.5 mM KCl was tested to evaluate the viability of rings. If maximal responses (*R*_MAX_) was lower than 80% of contraction obtained after CRC to K^+^, the artery was discarded.

### Solutions and Reagents

KRB contained (in mM) NaCl 118.5, NaHCO_3_ 25, KCl 4.7, KH_2_PO_4_ 1.2, MgSO_4_ 1.2, CaCl_2_ 2.5, glucose 5.5 with a pH of 7.4. In K-KRB (125 mM K^+^) NaCl was replaced by an equimolar amount of KCl. Noradrenaline, PHE, L-NAME, and prazosin were obtained from Sigma Chemical Co., USA.

### Analysis

Potency (EC_50_) and *R*_MAX_ to the different vasoactive agents tested were obtained by fitting the concentration-response curves to a Boltzmann function (Prism 4.0, Graphpad) as described previously (Moraga et al., 2011). *R*_MAX_ was expressed as tension (N/m) for K^+^ and/or as a percentage relative to a submaximal dose of 62.5 mM of KCl (expressed, %${\text{K}}_{\text{MAX}}^{+}$) for the adrenergic agonists. Values are normalized (%${\text{K}}_{\text{MAX}}^{+}$), in accord with sigmoidal shape and the model is assumed to have a standard slope with a Hill slope of 1.0 (Motulsky and Christopoulos, 2003). In this sense, potency was expressed as EC_50_ (the concentration at which 50% of *R*_MAX_ was obtained) for K^+^ or as pEC_50_ (–logEC_50_) for the adrenergic agonists. The relative affinity (pA_2_) for prazosin was estimated through Schild analysis by plotting log (*R* – 1) values for individual vessels against the antagonist (prazosin) concentration (log [A]), where *R* is defined as a ratio of the EC_50_ NA plus antagonist [A] divided by EC_50_ NAalone (Arunlakshana and Schild, 1959). Slope analysis was performed in each curve, supporting the presence of a competitive antagonist. Then, pA_2_ values were considered the *x*-intercept to the Schild slope (Motulsky and Christopoulos, 2003).

### Statistical Analysis

Data are expressed as means ± S.E.M. Two-way ANOVA for repeated measures followed by the *post hoc* test of Newman–Keuls or the Student's *t* test for unpaired data were used to compare data, as appropriate. Linear regression analysis of the Schild plot was used to estimate pA2 (intercept) values, the slope, and the correlation coefficient of the regression line (Motulsky and Christopoulos, 2003). For all comparisons, differences were considered significant when *p* < 0.05 (Zar, 1984).

## Results

### *Ex vivo* Small Femoral Arteries Wire Myography

#### Contractile Response to Potassium Chloride in Femoral Arteries of NB Llamas and Sheep of LA and HA

In the analysis of K^+^ CRC sensitivity, NBLL showed that HA neonates are more sensitive (EC_50_) than LA neonates. NBSH showed a similar potency (EC_50_) at LA and HA (Table 1). LA and HA NBSH potency was lower than LA and HA NBLL (*p* < 0.05) (Table 1; Supplementary Figure 1).

**Table 1:** Values of CRC in presence of potassium chloride, noradrenaline (NA), phenylephrine (PHE) in NBLL and NBSH at LA and HA.

	**NB Llama**		**NB Sheep**	
	**LA**	**HA**	**LA**	**HA**
**CCR K** ^**+**^				
EC_50_ (mM)	29.8 ± 1.6	25.1 ± 1.2 *	36.6 ± 1.7†	38.7 ± 3.7†
R_MAX_ (N/m)	15.6 ± 1.2	32.5 ± 0.5 *	10.6 ± 0.9†	8.3 ± 0.3*†
**CCR NA**				
pEC_50_ (M)	5.34 ± 0.13	6.51 ± 0.12 *	4.88 ± 0.28	5.61 ± 0.18*†
R_MAX_ (N/m)	16.2 ± 0.40	33.0 ± 0.60 *	13.8 ± 1.20	8.60 ± 0.20*†
R_MAX_ (%K^+^)	98.2 ± 3.50	101.5 ± 1.60	102.8 ± 1.60	105 ± 1.80
**CCR PHE**				
pEC_50_ (M)	5.52 ± 0.13	6.24 ± 0.14 *	4.72 ± 0.11†	6.08 ± 0.17*
R_MAX_ (N/m)	15.0 ± 0.30	30.1 ± 0.80 *	6.20 ± 0.23†	4.99 ± 0.21*†
R_MAX_ (%K^+^)	101.3 ± 2.3	95.4 ± 0.88	56.9 ± 1.10§†	57.9 ± 2.30§†

*Means ± SEM*;

* *p < 0.05 LA vs. HA*,

§ *p < 0.05 NA vs. PHE, and*

† *p < 0.05 NBLL vs. NBSH*.

Regarding the *R*_MAX_, LA NBLL was lower than HA NBLL (*p* < 0.05). In contrast with the NBLL, the LA NBSH had a greater maximal contraction than HA NBSH (*p* < 0.05) (Table 1). Moreover, LA and HA NBLL had a higher maximal contraction than LA and HA NBSH femoral vessels (*p* < 0.05) (Table 1).

#### Contractile Response to Adrenergic Agonists in Femoral Arteries of NB Llamas and Sheep of LA and HA

The absolute tension of HA-NBLL had a greater *R*_MAX_ to both NA and PHE than their LA counterparts (*p* < 0.05). However, when normalized to potassium, the *R*_MAX_ for NA and PHE were similar in LA- and HA-NBLL, but their corresponding pEC_50_ values were higher in HA- than LA-NBLL (*p* < 0.05) (Table 1). In NBSH, *R*_MAX_ for either NA or PHE expressed as absolute values were lower in HA- than LA-animals, but they were similar for K^+^-normalized values, while pEC_50_ was larger in HA- than in LA-NBSH (Table 1). When compared, the contraction to PHE was ~57% of the tension elicited by NA in NBSH (Table 1). Additionally, we observed that pEC_50_ and *R*_MAX_ to NA (in N/m) are similar between species at LA. However, the response to PHE of pEC_50_, *R*_MAX_ (N/m and %K^+^) in NBSH is lower than NBLL. When comparing both species at HA, we observed that pEC_50_ and *R*_MAX_ to NA (N/m and %K^+^) in NBSH were lower than NBLL (*p* < 0.05). In addition, the pEC_50_ in response to PHE was similar to NBLL and *R*_MAX_ (N/m and %K^+^) in NBSH and was lower than NBLL at HA (*p* < 0.05) (Supplementary Figure 2).

#### The Role of (NOS) and Endothelium in the Vasocontractile Response to PHE in NBLL and NBSH of LA and HA

**Figure 1:**
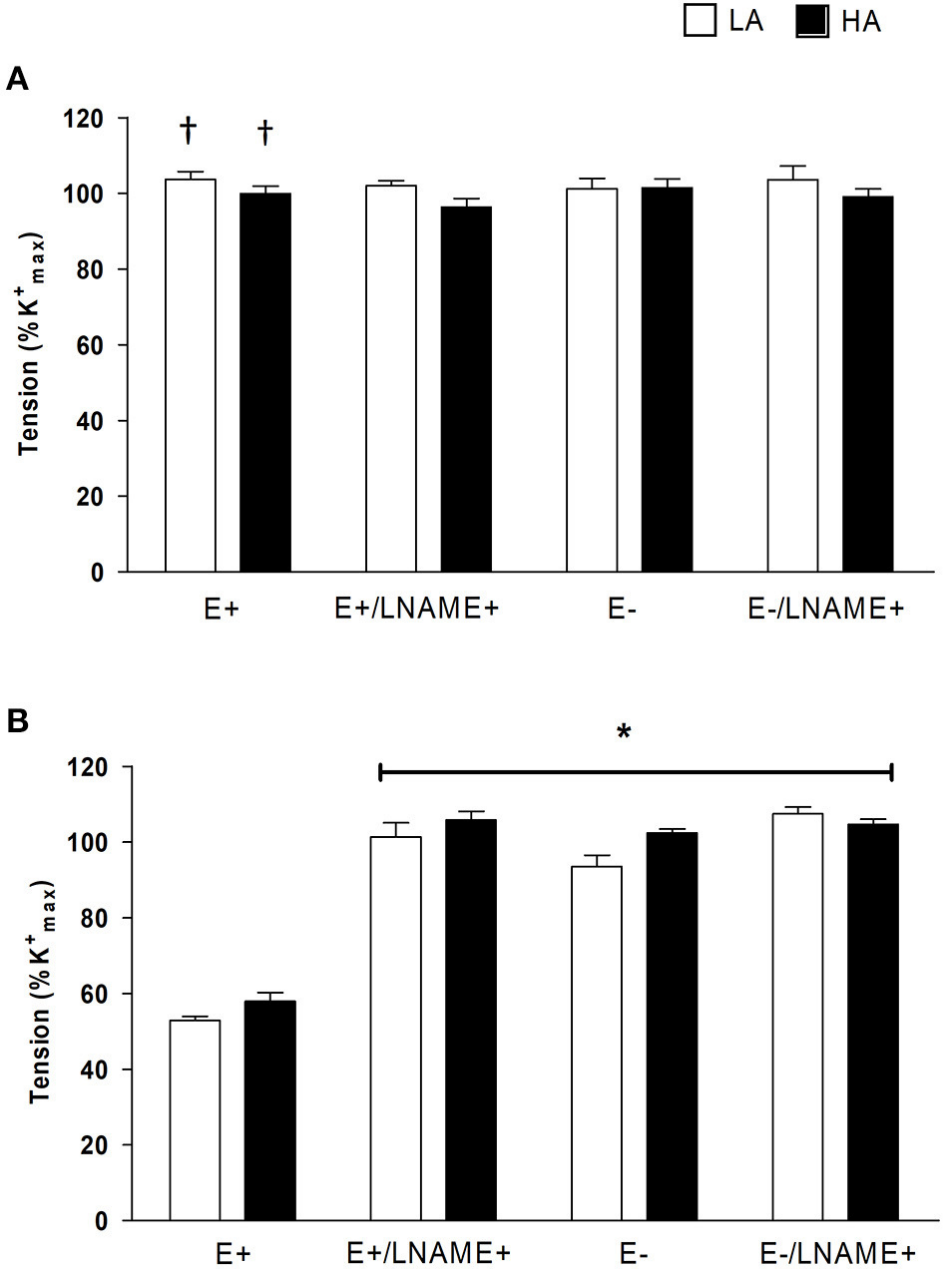
Effect of *N*G-nitro-l-arginine methyl ester L-NAME and endothelium remove (–E) on PHE-induced contraction. **(A)** In NBLL (*n* = 6) and CRC to PHE, and PHE after 30 min of incubation with 10^−5^ M L-NAME with endothelium (+E) and without endothelium (–E). **(B)** Effect of L-NAME on PHE-induced contraction in NBSH (*n* = 6) and CRC to PHE, and PHE after 30 min of incubation with 10^−5^ M L-NAME with endothelium (+E) and without endothelium (–E), where open bar represent LA and close bar represent HA. Values are expressed as means ± SEM. Asterisk (^*^) represent a significant difference between NBSH (LA/HA) PHE-induce contraction (+E) vs PHE-induce contraction (+E) plus L-NAME and PHE-induce contraction (–E) (*p* < 0.05). ^†^ Represent significant difference between NBLL vs NBSH (*p* < 0.05).

First, to probe the role of NOS we compared the CRC to PHE in the absence or the presence of NOS inhibition with L-NAME (10^−5^ M). In NBLL of either LA and HA, L-NAME did not modify *R*_MAX_ (Figure 1A; Table 2) or pEC_50_ of PHE (Table 2). In contrast, in NBSH of LA and HA, NOS blockade with L-NAME promoted an *R*_MAX_ of PHE similar to NA without modification in pEC_50_ (Figure 1B and Table 2). To examine the role of endothelium, we denudated small femoral arteries in both species and performed CRC again for PHE in the presence and the absence of L-NAME. In NBLL of either LA or HA, denudation had no effect on pEC_50_ or *R*_MAX_ to PHE (Figure 1A and Table 2). In contrast, in NBSH of LA and HA, denudation of small femoral arteries increased the *R*_MAX_ for PHE, with values similar to those in the presence of NA without modification in pEC_50_ (Figure 1B and Table 2). Lastly, we determined the role of NOS blockade with L-NAME (10^−5^ M) and arterial denudation in both species. Again, we performed CRC for PHE. In the NBLL of either LA or HA, denudation did not affect pEC_50_ or *R*_MAX_ to PHE (Figure 1A and Table 2). NBSH of LA and HA, blocked and with denudation of small femoral arteries, increased *R*_MAX_ for PHE to values similar to those observed in the presence of NA without modification in pEC_50_ (Figure 1B, Tables 1, 2). When comparing both species at LA, we observed that pEC_50_ in NBLL was higher than NBSH in all experimental conditions. However, when comparing both species at HA, values of pEC_50_ in NBLL were similar to NBSH (Supplementary Figure 3).

**Table 2:** Effect of L-NAME blocked and denudation of small femoral arteries in NB LL and NBSH at LA and HA.

	**NB llamas**		**NB Sheep**	
	**LA**	**HA**	**LA**	**HA**
**CRC PHE (+E)**				
p_EC50_ (M)	5.43 ± 0.17	6.48 ± 0.15 ^*^	4.56 ± 0.04^†^	6.08 ± 0.17^*^
R _MAX_(%K^+^)	103.7 ± 2.00	105.5 ± 1.60	52.8 ± 1.10^§^^†^	57.9 ± 2.30^§^^†^
**CRC PHE (–E)**				
p_EC50_ (M)	5.89 ± 0.15	6.68 ± 0.17^*^	4.88 ± 0.08^†^	6.00 ± 0.12^*^
R _MAX_(%K^+^)	101.2 ± 2.80	101.5 ± 1.60	96.5 ± 2.2	105.4 ± 1.10
**CRC PHE (+E)+LNAME**				
p_EC50_ (M)	5.90 ± 0.14	6.24 ± 0.19	4.85 ± 0.18^†^	5.93 ± 0.15^*^
R_MAX_ (%K^+^)	102.0 ± 1.30	98.4 ± 2.20	94.9 ± 2.10^†^	102.0 ± 1.30
**CRC PHE (–E)+LNAME**				
p_EC50_ (M)	5.97 ± 0.17	6.17 ± 0.15	4.47 ± 0.17^†^	6.08 ± 0.12^*^
R _MAX_ (%K^+^)	103.6 ± 3.60	99.4 ± 2.20	102.3 ± 3.50	104.7 ± 1.30

*Mean ± SEM*;

* *p < 0.05 LA vs HA*;

§ *p < 0.05 Phe (+E) vs (–E), (+ L-NAME) and (–E +L-NAME)*;

† *p <0.05 NBLL vs NBSH*.

#### Relative Affinity (pA_2_) to NA in Femoral Arteries in NB Llama and Sheep at LA and HA

**Figure 2:**
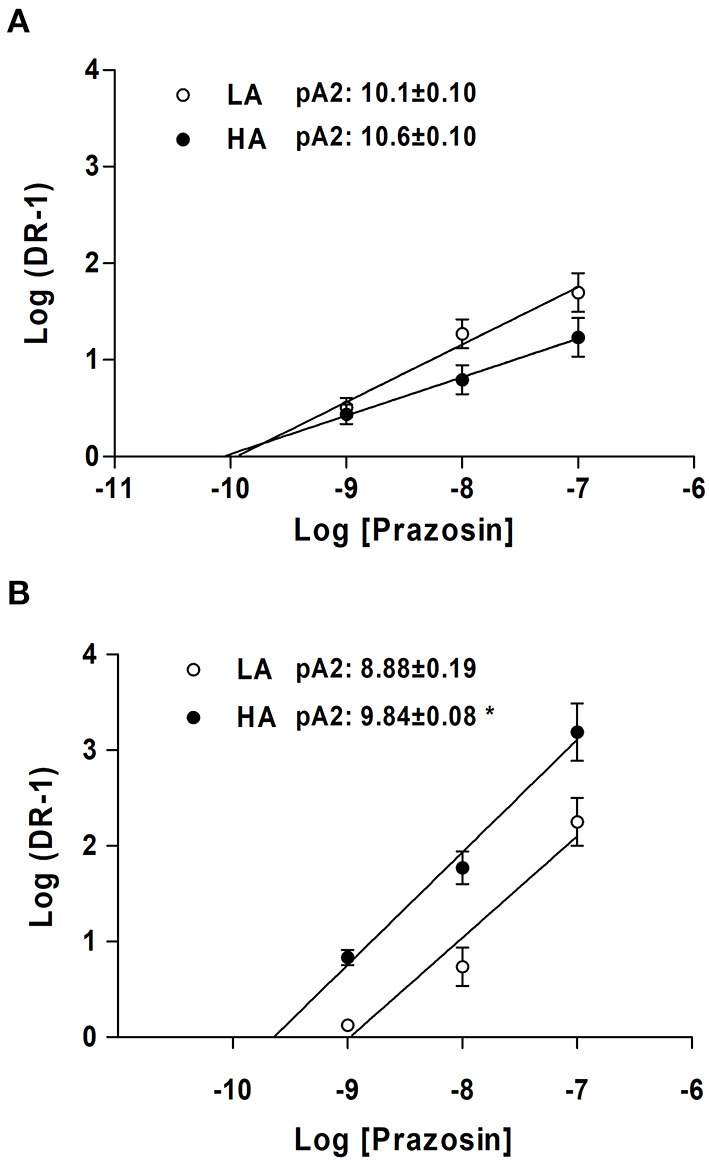
Relative affinity (pA_2_) in NBLL and NBSH of LA and HA obtained from concentration response curves to NA and NA in the presence of prazosin at 1, 10, and 100 nM and Schild plots analysis. **(A)** NBLL at LA vs HA and **(B)** NB sheep. Values are expressed as means ± SEM. Asterisk (*) represent a significant difference between NBSH (LA/HA) (*p* < 0.05).

The blockade with prazosin, a selective α_1_-adrenergic antagonist, promoted a concentration-dependent rightward shift of pEC_50_ for NA, in both LA- and HA-NBLL. This rightward shift is consistent with competitive blockade (Supplementary Figure 4). Competitive inhibition experiments modified *R*_MAX_ for NA, except at the highest concentration tested in NBLL at LA compared with HA. The Schild plot analysis did not show changes in pA_2_ values between LA- and HA-NBLL, suggesting that the affinity of the α-adrenergic receptor for prazosin was unchanged (Figure 2A). In both LA- and HA-NBSH, prazosin blockade also promoted a rightward shift of pEC_50_ for NA as prazosin concentration increased. Increasing prazosin concentration did not decrease *R*_MAX_ for NA except at the highest concentration tested in NBSH at HA. However, the Schild analysis showed a greater pA_2_ value in HA- than LA-NBSH, consistent with a greater affinity of the α-adrenergic receptor for prazosin at HA (Figure 2B). Further, when comparing both species at LA and HA, we observed that NBLL values for pA_2_ were greater than those obtained at LA in NBSH (*p* < 0.05). However, no difference was observed in NBLL and NBSH at HA.

## Discussion

We found that HA chronic hypoxia-induced enhanced femoral α-adrenergic vascular reactivity in NBLL compared with NBSH.

The enhanced α-adrenergic vascular reactivity found in the HA NBLL is also observed in the fetal and neonatal llama at sea level (Giussani et al., 1999; Moraga et al., 2011; Reyes et al., 2020). The importance of the α-adrenergic signaling during acute hypoxia in the fetus is vital since its blockade causes cardiovascular collapse and death (Giussani et al., 1999).

Our previous work has shown that intense peripheral vasoconstriction is essential for blood flow redistribution in response to acute hypoxia in the llama fetus and that α-adrenergic signaling is necessary for eliciting this response which is critical for fetus survival (Giussani et al., 1999). As indicated, this high peripheral vasoconstrictor tone persists in the neonatal period (Reyes et al., 2018, 2020). Moreover, we described the high femoral vascular resistance either basally or under acute hypoxia in LA-NBLL compared with LA-NBSH (Moraga et al., 2011). We also described that this higher femoral vascular resistance in the LA-NBLL could be explained by a powerful α_1_ adrenergic tone and the preferential expression of the α_1B_ adrenoceptor subtype. In contrast, the LA-NBSH preferentially expressed the α_1A_ adrenoceptor (Moraga et al., 2011). Our finding of a greater R_MAX_ to NA and PHE in HA-NBLL compared with HA-NBSH llamas are consistent with higher α_1B_ adrenoceptor subtype levels in the former.

In the present study, we have extended our previous work and have now compared the effect of HA chronic hypoxia in the femoral vasoconstrictor response in two species, llama and the sheep, with different ancestry at HA.

### Vasoconstriction Mediated by Potassium in NBLL and NBSH at LA/HA

#### Maximum Response to Potassium Chloride

Regarding increased *R*_MAX_ for K^+^ observed in animals with chronic exposure like NBLL at HA, there are no previous studies published on the effect of hypobaric hypoxia on femoral vessels in the NBLL. Exposure of rats to hypoxia of 10% by 24 h and 48 h decreases the contractile response in aorta rings (Auer and Ward, 1998), while pulmonary arterial bed hypoxia promotes vasoconstriction, upregulation of voltage-dependent Ca^2+^ channels, and a hyperplasic/hypertrophic structural remodeling related with increased *R*_MAX_ (Wan et al., 2013; Dunham-Snary et al., 2017; Reyes et al., 2018; Gassmann et al., 2021). Other mechanisms such as increased Ca^2+^ sensitization in vascular smooth muscle can also increase *R*_MAX_ (Jernigan and Resta, 2014). To the best of our knowledge, hypoxic remodeling, an increase of Ca^2+^ sensitization, or upregulation of voltage-dependent Ca^2+^ channels have not been described in femoral vessels; nevertheless, we cannot rule out these possibilities. The mechanism involved in the increase of femoral *R*_MAX_ to K^+^ in HA-NBLL may improve the total peripheral contraction capacity and blood flow redistribution to withstand hypoxia in this species.

In contrast, the decreased *R*_MAX_ described in our study in small femoral arteries NBSH at HA is in agreement with previous observations in the same ovine neonatal model (Herrera et al., 2007) and in the carotid artery in fetal sheep (Nauli et al., 2005). Decreased sensitivity of the contractile smooth muscle machinery to Ca^2+^ could explain our results (Nauli et al., 2005). The highest function or expression of vasodilator mechanisms, like nitric oxide signaling, cannot be excluded in NBSH at HA (Herrera et al., 2008, 2019; Reyes et al., 2018).

#### Potency to Potassium (EC_50_)

NBLL responses increased potency at HA compared with LA. Meanwhile, NBSH showed similar potency values at LA and HA. However, there was a larger potassium potency (low values of EC_50_) in NBLL at LA or HA than NBSH at LA and HA. In agreement with Nernst Equation, the lower EC_50_ values to K^+^ described in NBLL means that it can activate a vasocontraction at a lower membrane potential (see Papamatheakis et al., 2012). This suggests that NBLL, as a species, adapted to chronic HA hypoxia over millions of years, has developed a lower threshold for calcium channel type-L activation (Ghosh et al., 2017; Ottolini et al., 2019).

### Vasoconstriction Mediated by α_1_-Adrenoreceptors in NBLL and NBSH at LA/HA

#### NBLL at LA and HA

Our study depicted the outcomes of chronic exposure at HA on vasoconstriction mechanisms mediated by α_1_-adrenoceptors in NBLL compared with LA-NBLL. Vasocontraction mediated by NAor PHE in NBLL are similar to those reported previously in NBLL at LA (Moraga et al., 2011). We observed a similar pattern in NBLL at HA, resulting in increased vasocontraction when HA-NBLL was compared with LA-NBLL. Furthermore, an increased pEC_50_ was described in NBLL at HA. We did not observe any potentiation in the PHE vasoconstrictor effect using L-NAME or femoral artery denudation in LA- or HA-NBLL. This evidence supports the presence of α_1−_adrenoceptors in small femoral arteries as the dominant mechanism accounting for the powerful vasoconstrictor tone in NBLL from HA or LA.

To characterize the α_1_-adrenoceptor we performed competitive inhibition with increased doses of prazosin followed by CRC to NA. Schild analysis performed with NBLL showed similar pA_2_ value for both LA- and HA-NBLL, indicating the presence of one α_1−_adrenoceptor subtype under both conditions. In addition, pA_2_ > 9 value, which suggests the presence of a receptor with a high affinity for prazosin (Graham et al., 1996; Moraga et al., 2011; Docherty, 2019). Nevertheless, competitive blockade was performed using prazosin, a specific α_1_-adrenoceptor blocker. Therefore, the difference observed in potency to NA and PHE at HA could be explained by the expression of other α_1_-adrenoceptor subtypes. NBLL at LA have preferential expression of the α_1B_-adrenoceptor subtype over the α_1A_-adrenoceptor subtype transcript (Moraga et al., 2011). However, further studies using specific subtype α_1_-adrenoceptor blockers are required to evaluate subtype expression in NBLL at HA appropriately.

#### NBSH at LA/HA

*R*_MAX_ and pEC_50_ values for the α-adrenergic agonist (NA and PHE) described in small femoral arteries in the present work are similar to values reported in NBSH at LA (Herrera et al., 2008; Moraga et al., 2011). However, NBSH exposed to HA showed increased potency and reduced *R*_MAX_ to NA and PHE. The *R*_MAX_ values in response to PHE in NA at LA and HA show the same tendency to previously described results in NBSH at LA (Moraga et al., 2011). We can discard the vasoconstrictor role of α_2−_adrenoceptor since clonidine does not promote vasoconstriction in small femoral arteries nor at LA or HA in the present study (Moraga et al., 2011). To demonstrate that α_1_-adrenoceptor could promote NO production by attenuating vasoconstriction at close to 50%, we studied the contraction under NOS inhibition (L-NAME 10^−5^ M) or after removing the endothelium, both conditions permit 100% vasoconstriction recovery with PHE, similar to that observed with NA in NBSH at LA and HA. A similar pattern was described in the bronchial resistance artery in rabbits (Zschauer et al., 1997) and mesenteric resistances artery in rats (Dora et al., 2000). The proposed mechanism suggests that PHE promotes α_1_-adrenoceptor activation in endothelium, increasing NO production that opposes vasoconstriction (Tuttle and Falcone, 2001; Raj and Subramani, 2016; Marconi et al., 2020). This result suggests different subtypes of α_1_-adrenoceptor with a specific location in the small femoral artery, one type of α_1_-adrenoceptor present in the endothelium, and other α_1_-adrenoceptors present in smooth muscle cells, as proposed by Moraga et al. (2011).

To characterize the α_1_-adrenoceptor, we performed competitive inhibition experiments with increasing doses of prazosin followed by CRC to NA. Schild analysis performed to NBSH showed two different pA_2_ values, suggesting a low-affinity receptor in LA-NBSH, similar to previous reports (Moraga et al., 2011) and according to the nomenclature proposed by Graham et al. (1996) and Docherty (2019). Also, the pA_2_ > 9 value suggests the presence of a receptor with a high affinity for prazosin in HA-NBSH (Graham et al., 1996; Moraga et al., 2011; Docherty, 2019). These outcomes indicate the presence of two α_1−_adrenoceptor subtypes in small femoral arteries at LA and HA-NBSH. Nowadays, low-affinity phenotype has been associated with the subtype α_1A_ adrenoceptor (Docherty, 2019). Accordingly, we previously demonstrated that NBSH at LA preferentially expresses subtype α_1A_-adrenoceptor (Moraga et al., 2011).

In conclusion, the *ex vivo* results obtained from small femoral arteries of NBLL and NBSH gestated at HA showed that the physiology underlying this difference between species is due to the following (1) enhanced vasoconstrictor reactivity and potency to α-adrenergic agonists in NBLL; (2) an important role of NO and endothelium in NBSH in the modulation of vasoconstriction induced by α_1_-adrenergic stimuli, and (3) maintenance in pA_2_ in NBLL at LA/HA. In NBSH, increased pA_2_ was observed at HA respect LA.

## Data Availability Statement

The original contributions presented in the study are included in the article/Supplementary Material, further inquiries can be directed to the corresponding author.

## Ethics Statement

The animal study was reviewed and approved by Faculty of Medicine University of Chile.

## Author Contributions

FM, RR, and AL conceived and designed the study. FM, GE, and VL supervised the overall study. FM and GE contributed to sample data collections and statistical analysis. All authors drafted the report and contributed to the interpretation of the results, critical revision of the manuscript, and approval of the final manuscript. FM is the guarantor.

## Conflict of Interest

The authors declare that the research was conducted in the absence of any commercial or financial relationships that could be construed as a potential conflict of interest.

## Publisher's Note

All claims expressed in this article are solely those of the authors and do not necessarily represent those of their affiliated organizations, or those of the publisher, the editors and the reviewers. Any product that may be evaluated in this article, or claim that may be made by its manufacturer, is not guaranteed or endorsed by the publisher.
