# Supplementary material for: Enhanced Vasoconstriction Mediated by α1-Adrenergic Mechanisms in Small Femoral Arteries in Newborn Llama and Sheep Gestated at Low and High Altitudes
Source: Front Physiol. 2021 Aug 4;12:697211. doi: 10.3389/fphys.2021.697211 (PMC8371382; doi:10.3389/fphys.2021.697211)
Supplement: Supplementary file 1 [file Table_1.DOCX]

| Supplementary Table .- Characteristics of the sample | | | | |
| --- | --- | --- | --- | --- |
|  | Low Altitude (LA) | | High Altitude (HA) | |
|  | NB llama | NB sheep | NB llama | NB sheep |
| Numbers (n) | 7 | 8 | 6 | 6 |
| Weight (Kg) | 12.0 ± 1.4 | 8.7 ± 1.5 | 9.0 ± 1.0 | 6.7 ± 0.8 |
| Age (Days) | 12.0 ± 1.0 | 13.0 ± 1.0 | 12.0 ± 1.0 | 13.0 ± 1.0 |
| Gender (M/F) | 4/3 | 5/3 | 3/3 | 3/3 |
| Optimal diameter (μm) | 350 ± 30 | 359 ± 33 | 325 ± 50 | 315 ± 43 |
| Vessel length (mm) | 1.8 ± 0.1 | 1.8 ± 0.1 | 1.9 ± 0.1 | 1.9 ± 0.1 |
|  |  |  |  |  |
| Values expressed means ± SEM | | | | |
