# Supplementary material for: Enhanced Vasoconstriction Mediated by α1-Adrenergic Mechanisms in Small Femoral Arteries in Newborn Llama and Sheep Gestated at Low and High Altitudes
Source: Front Physiol. 2021 Aug 4;12:697211. doi: 10.3389/fphys.2021.697211 (PMC8371382; doi:10.3389/fphys.2021.697211)
Supplement: Supplementary file 2 [file Data_Sheet_1.PDF]

Supplementary Figures

Legends figures

Figure 1.- Concentration response curves to potassium chloride in small femoral arteries of newborn llama (NBLL) and newborn sheep (NBSH) gestated at Low Altitude (LA, open circle) and High Altitude (HA, close circle). Each point represents a means of tension expressed in (N/m) and brackets represent +SE.

Figure 2.- Concentration response curves to Noradrenaline (NA) and Phenylephrine (PHE) in small femoral arteries of newborn llama (NBLL, A and B) and newborn sheep (NBSH, C and D) gestated at Low Altitude (LA, open circle) and High Altitude (HA, close circle). Each point represents a means of tension expressed in % K max and brackets represent +SE.

Figure 3.- Effect of endothelium remove (-E) and *N*G-nitro-L-arginine methyl ester (L-NAME, 10^{-5} M) on PHE-induced contraction. A) NBLL at LA, B) NBLL at HA, C NBSH at LA and D) NBSH at HA. Open circles represent arteries with endothelium (+E) and open triangle represent arteries without endothelium (-E), close circle represents arteries +E plus L-NAME and close triangle represent arteries (-E) plus L-NAME. Each point represents a means of tension expressed in (N/m) and brackets represent +SE.

Figure 4.- Effect of prazosin on NA-induced contraction in small femoral arteries of NBLL (LA, A and HA, B) and NBSH (LA, C and HA, D). Concentration response curves to NA (control, open circle) and NA in presence of prazosin 1 nM (close circle), 10 nM (close triangle), and 100 nM (close rhombus). Each point represents a means of tension expressed in (N/m) and brackets represent +SE.

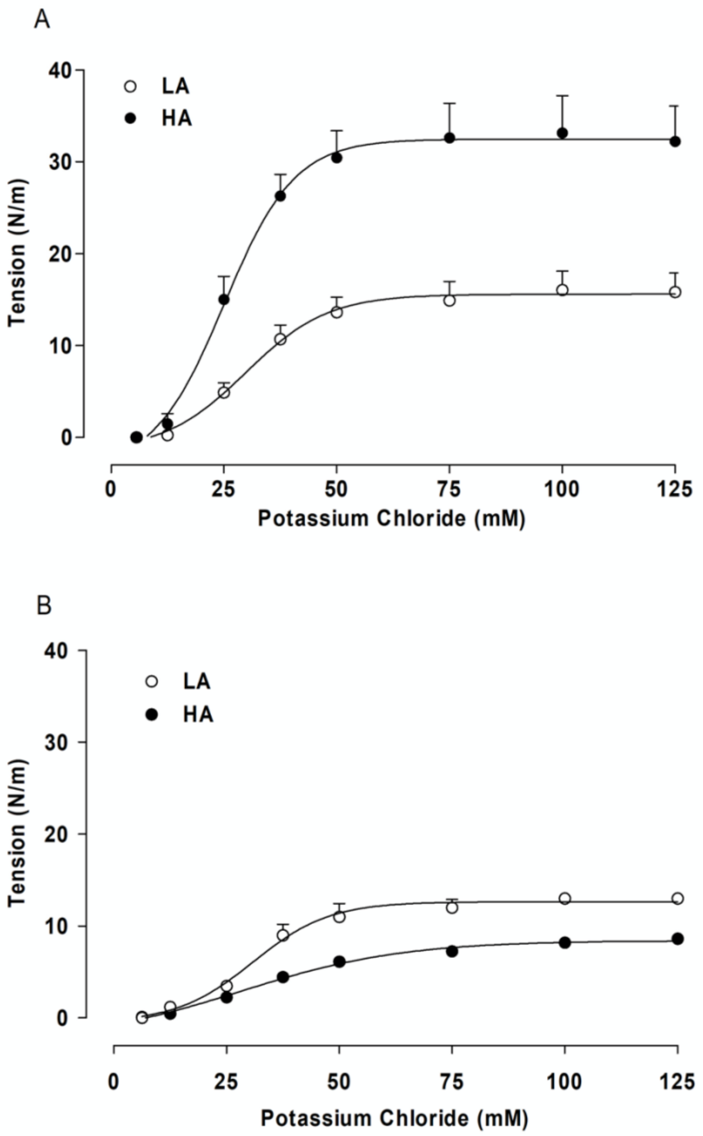

Supplementary Figure 1

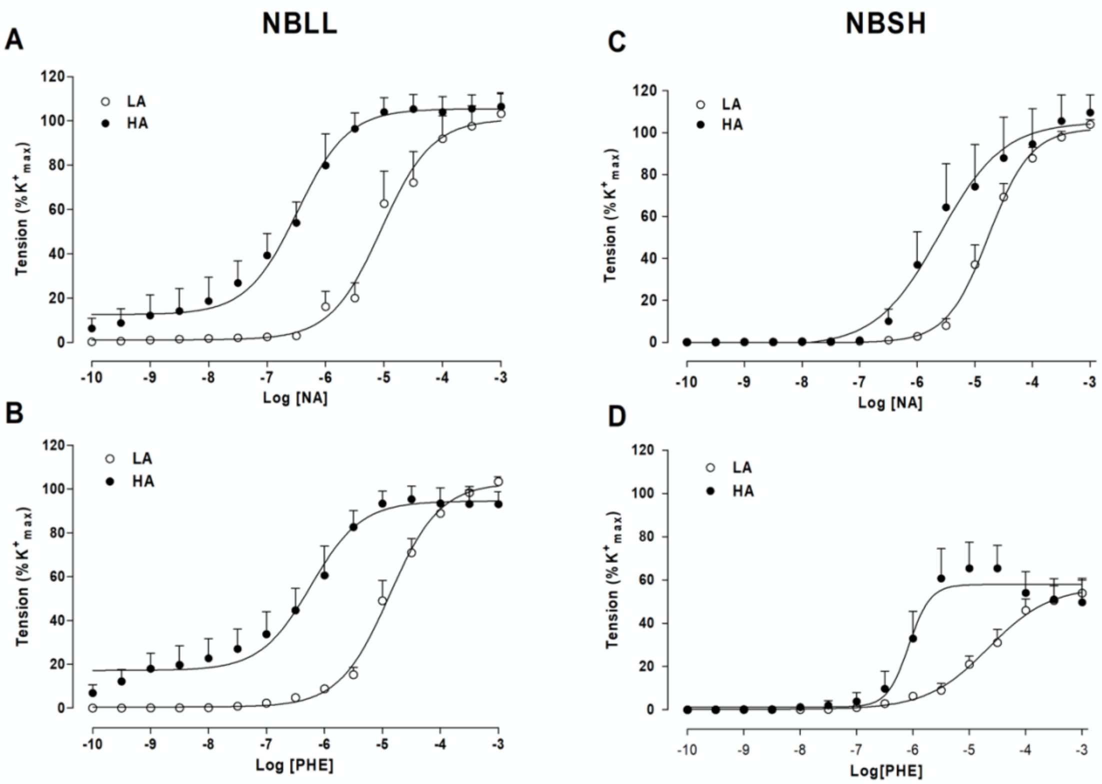

Supplementary figure 2

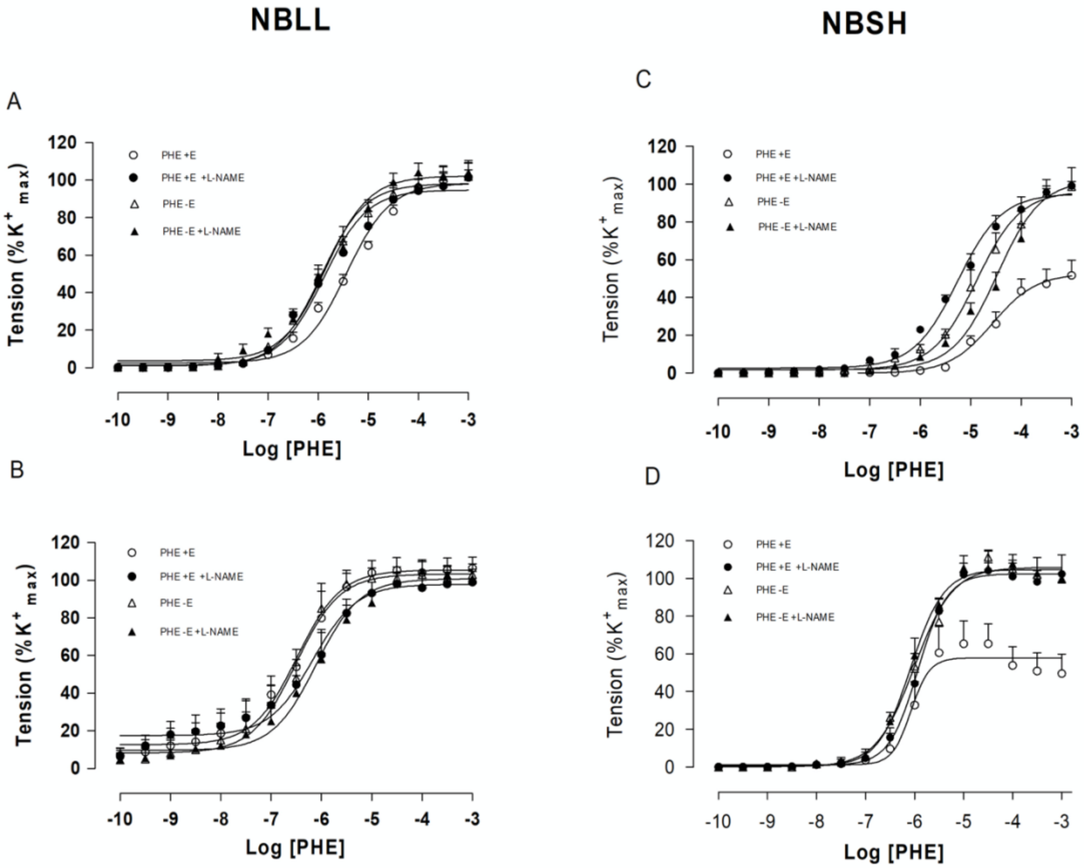

Supplementary Figure 3

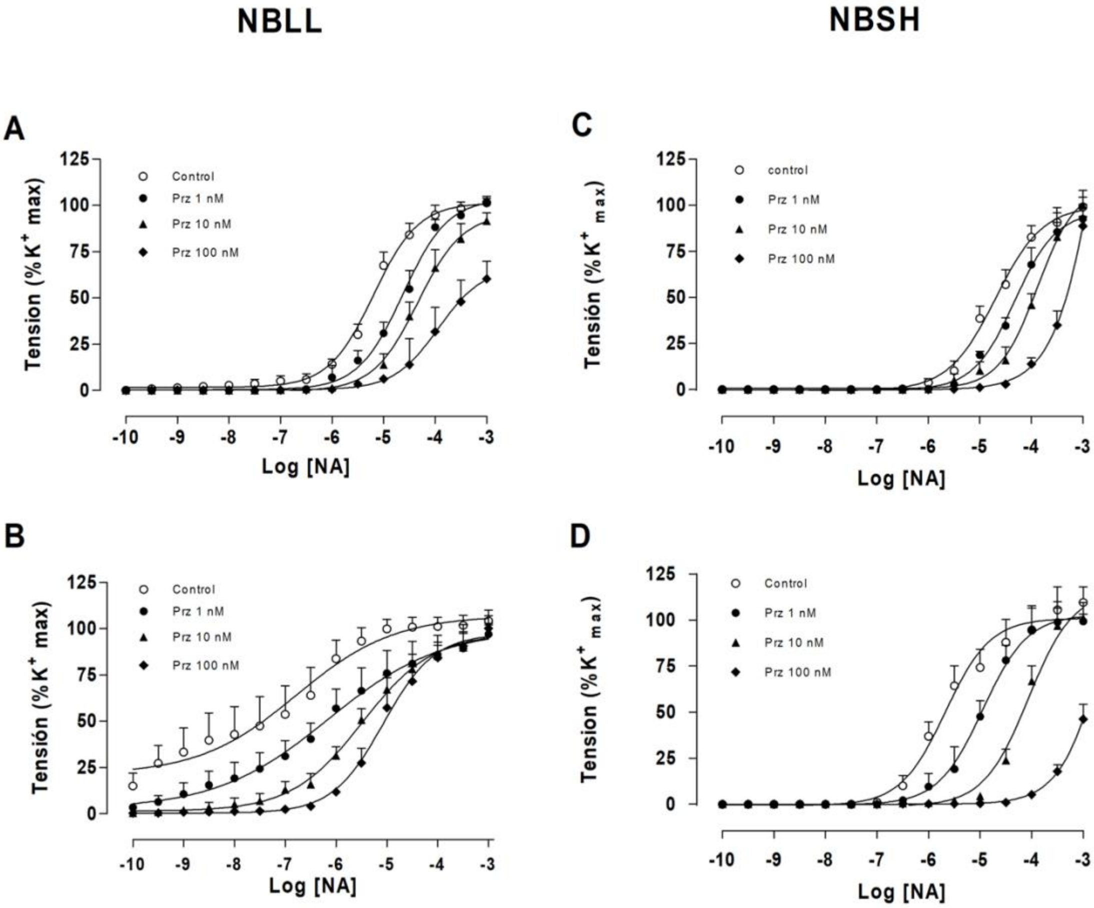

Supplementary Figure 4
